# Poor outcome associated with mucormycosis in critically ill hematological patients: results of a multicenter study

**DOI:** 10.1186/s13613-021-00818-4

**Published:** 2021-02-10

**Authors:** Matthieu Jestin, Elie Azoulay, Frédéric Pène, Fabrice Bruneel, Julien Mayaux, Martin Murgier, Michael Darmon, Sandrine Valade

**Affiliations:** 1 Service de Médecine Intensive Et Réanimation, Hôpital Saint-Louis, 1 Avenue Claude Vellefaux, 75010 Paris, France; 2 Université de Paris, 85 Boulevard Saint-Germain, 75006 Paris, France; 3 Service de Médecine Intensive Et Réanimation, AP-HP, Hôpital Cochin, 27 Rue du Faubourg Saint-Jacques, 75014 Paris, France; 4 Service de Réanimation Médico-Chirurgicale, Centre Hospitalier de Versailles, 177 Rue de Versailles, 78150 Le Chesnay, France; 5 Service de Pneumologie, Médecine Intensive Et Réanimation, Hôpital Universitaire Pitié-Salpêtrière, 47-83 Boulevard de l’Hôpital, 75013 Paris, France; 6 Service de Réanimation Polyvalente, Centre Hospitalo-Universitaire de Saint-Etienne, 25 Boulevard Pasteur, 42055 Saint-Etienne, France

**Keywords:** Mucormycosis, Intensive care unit, Hematological malignancies, Outcome, Bone marrow transplantation

## Abstract

**Background:**

Mucormycosis is an emerging fungal infection that may lead to multi-organ failure, especially in patients with hematological malignancies (HM). We performed a retrospective, cohort study, in five intensive care units (ICU) to assess the outcome of critically ill patients with HM and mucormycosis between 2002 and 2018. The secondary objective was to identify prognostic factors in this setting.

**Results:**

Twenty-six patients were included with a median age of 38 years [IQR, 26–57]). Acute leukemia was the most frequent underlying disease (50%). Nine patients (35%) underwent allogeneic stem cell transplantation (SCT). Nineteen patients (73%) had neutropenia and 16 (62%) had received steroids. The main reason for admission was acute respiratory failure (*n* = 14, 54%) followed by shock (*n* = 5 19%). The median SOFA score at admission was 7 [5–8].

According to EORTC/MSG criteria, mucormycosis was "proven" in 14 patients (54%), "probable" in 5 (19%) and “possible” in 7 (27%) in whom diagnosis was made by qPCR. *Rhizopus* and *Mucor* were the most frequent documented species. Seven patients (27%) had concurrent *Aspergillus* infection. Mucormycosis was diagnosed 1 day [−4 to + 6] after ICU admission. Sixteen patients (62%) had pulmonary involvement and ten (38%) rhino-cerebral involvement. Infection was disseminated in eight patients (31%). Twenty-two patients (85%) were treated with liposomal amphotericin B; 12 (46%) received antifungal combination including posaconazole in 7. Eight patients (31%) underwent curative surgery. Twenty-one patients (81%) required invasive mechanical ventilation (IMV), 18 (69%) vasopressors, and 9 (35%) renal replacement therapy. ICU and hospital mortality rates were 77% and 88%, respectively. The median overall survival was 9 days [3–22]. IMV was strongly associated with ICU mortality (*p* < 0.001) Three variables were associated with day 90 mortality in a Cox model including allogeneic SCT (HR 4.84 [95% CI 1.64–14.32]), SOFA score (1.19 [1.02–1.39]) and dual therapy (3.02 [1.18–7.72]).

**Conclusions:**

Mucormycosis is associated with a high mortality rate in patients with HM, especially in allogeneic SCT recipients. Benefit of ICU management in these patients should be assessed before admission and strategies aiming to improve these patients’ outcome are urgently needed.

## Background

Mucormycosis is an emerging fungal infection whose incidence has increased by 7.3% per year between 2001 and 2010 [1]. Hematological patients with profound neutropenia [2] or allogeneic hematopoietic stem cell transplant (SCT) recipients are at high risk of mucormycosis and count for half of reported cases [3]. In the context of allogeneic SCT, graft-versus-host disease (GVHD), especially if treated with steroids, is a well-established risk factor for mucormycosis [4]. The increasing rate of invasive mucormycosis, although its true incidence remains underestimated, is multifactorial. First, new immunosuppressive therapies have been developed and their use increased the number of patients at higher risk of mucormycosis. Moreover, the large use of antifungal agents induces selection pressure and progressively increases the risk of developing the disease. Finally, the development of new diagnostic tools enhances ability to detect this later [5].

Invasive mucormycosis can lead to multi-organ failure which requires intensive care unit (ICU) management. While invasive fungal infections (IFI) are associated with a poor outcome in ICU patients with hematological malignancies (HM), most of the available data arise from series reporting invasive aspergillosis [6] and data regarding critically ill patients with mucormycosis are limited. The objective of our study was to assess the outcome of patients with HM admitted to the ICU for the management of invasive mucormycosis.

## Methods

### Study design and cohort

We performed a multicenter, retrospective, observational cohort study conducted in five ICUs in France. All adult patients (≥ 18 years) with HM and/or allografts consecutively admitted to the ICU between January 1, 2002 and December 31, 2018 with an ongoing invasive mucormycosis were included. Mucormycosis was defined as either a proven or probable infection according to EORTC/MSG criteria [7] or, a possible diagnosis of mucormycosis only if associated with positive Mucorales quantitative polymerase chain reaction (qPCR). Only first ICU hospitalization was considered in the analysis.

The primary objective was to assess the outcome of patients with HM admitted to ICU with invasive mucormycosis. Survival censored at day 90 was the main judgment criterion. The secondary objective was to assess prognostic factors.

### Data collection and definitions

Data reported in tables and figures were abstracted from the medical records. Clinical and laboratory data at ICU admission were collected, as well as organ failure and specific management during ICU stay. SOFA score was applied to assess severity at admission according to organ failures [8]. ICU and hospital mortality were available for all patients.

Neutropenia was defined as neutrophils count < 500/mm^3^ or white blood cells count < 1.000/mm^3^. Mucormycosis were considered disseminated if ≥ 2 distinct organs were involved. Sino-orbital and rhino-cerebral involvements were considered as one. Acute respiratory failure was defined by tachypnea > 30/min, respiratory distress, SpO2 < 90% at ICU admission and/or labored breathing [9]. Sepsis was established according to the 2001 task force definitions [10].

### Ethical considerations

The ethical committee of the French Society of Intensive Care has reviewed and approved the project (number CE SRLF 19–12). In accordance with the French legislation, the database was declared to the CNIL (“Commission Nationale de l’Informatique et des Libertés”) (number 2211255 v 0).

### Statistical analysis

Survival rates were established by the Kaplan–Meier method. For subgroup comparisons, Fisher’s exact test was used for binary variables and Mann–Whitney for continuous variables. Independent risk factors of day 90 mortality were assessed using Cox model. Conditional stepwise variable selection was performed with 0.2 as the critical P value for entry into the model, and 0.1 as the *p* value for removal. It was planned, should this variable not be selected, to force one by one, surgery or use of dual antifungal therapy during ICU stay, should these variables not be selected. Interactions and correlations between the explanatory variables were carefully checked. Validity of proportional hazards assumption, influence of outliers, and linearity in relationship between the log hazard and the covariates were carefully checked. All tests were two-sided, and P values less than 0.05 were considered statistically significant. Analyses were done using R software version 3.6.2 (https://www.r-project.org), including ‘survival’ package.

## Results

Twenty-six patients were included with a median age of 38 years [IQR, 26–57], half of them (*n* = 13) being of male gender (Table 1). Median Charlson score was 2 [IQR, 2–3]. Acute leukemia was the most frequent underlying disease (*n* = 13, 50%). Eight patients (31%) were recently diagnosed with HM while 11 (42%) had at least a partial response under treatment and 5 (19%) had a progressive disease. Patients had received 1 line of antitumoral treatment [IQR, 1–2], including autologous SCT in 4 patients. Nine patients (35%) were allogeneic SCT recipients. Nineteen patients (73%) had neutropenia at mucormycosis diagnosis. During the last 3 months, 16 patients (62%) had received steroids, mostly for HM (*n* = 11) or GVHD (*n* = 4).

**Table 1:** Characteristics of patients at ICU admission

N (%) or Median (IQR)	All n = 26	Survivors in ICU n = 6	Non survivors in ICU n = 20	*P* value
***Demographics***				
Age (years)	38 [26–57]	28 [34–35]	44 [28–58]	0.26
Male gender	13 (50)	3 (50)	10 (50)	1.00
***Hematological malignancy***				0.78
Acute leukemia	13 (50)	3 (50)	10 (50)	
- ALL	7	2	5	
- AML	6	1	5	
Lymphoma	5 (20)	1 (17)	4 (20)	
- Hodgkin	1	0	1	
- Non-Hodgkin	4	1	3	
Aplastic anemia	4 (15)	1 (17)	3 (15)	
Other^a^	4 (15)	1 (17)	3 (15)	
***Status of HM***				0.41
Recently diagnosed	8 (31)	3 (50)	5 (25)	
At least partial response	11 (42)	1 (17)	10 (50)	
Progression	5 (19)	2 (33)	3 (15)	
Unknown	2 (8)	0	2 (10)	
***Previous treatments***				
Allogeneic SCT	9 (35)	0	9 (45)	0.12
Steroids during the last 3 months	16 (62)	5 (83)	11 (55)	0.52
Antifungal prophylaxis	7 (27)	3 (50)	4 (22)	0.44
***Reason for ICU admission***				0.15
Acute respiratory failure	14 (53)	2 (33)	12 (60)	
Shock	5 (19)	3 (50)	2 (10)	
Perioperative management	3 (12)	0	3 (15)	
Coma	1 (4)	0	1 (5)	
Acute kidney injury	1 (4)	0	1 (5)	
Other^b^	2 (8)	1 (17)	1 (5)	
***Biological tests***				
Creatinine (µmol/L)	84 [49–119]	76 [56–90]	86 [45–123]	0.46
Albumin (g/L)	26 [23–31]	28 [24–30]	25 [23–30]	0.85
Bilirubin (µmol/L)	25.9 [15.0–43.0]	25.1 [17.1–30.8]	25.9 [12.7–46.5]	1.00
Leukocytes (G/L)	0.22 [0.10–2.60]	0.58 [0.21–1.06]	0.20 [0.10–4.30]	0.85
Hemoglobin (g/dL)	8.4 [7.9–9.1]	8.0 [7.1–9.5]	8.5 [8.0–9.0]	0.50
Platelets (G/L)	25 [13–42]	17 [6–30]	26 [14–41]	0.24
Prothrombin (%)	70 [50–76]	56 [48–73]	71 [58–75]	0.55
SOFA score	7 [5–8]	6 [5–6]	7 [5.5–9.5]	0.18
***Treatments in the ICU***				
Mechanical ventilation	21 (81)	1 (17)	20 (100)	* < 0.001*
Vasopressors	18 (69)	3 (50)	15 (75)	0.51
Renal replacement therapy	9 (35)	0	9 (45)	0.12

*ICU* intensive care unit, *ALL* acute lymphoblastic leukemia, *AML* acute myeloid leukemia, *HM* hematological malignancy, *SCT* stem cell transplantation, *SOFA score* Sepsis-related Organ Failure Assessment

^a^Myelodysplastic syndrome, chronic myeloid leukemia, hairy cell leukemia and hemophagocytic syndrome

^b^Including diabetic ketoacidosis and monitoring

The main reason for ICU admission was acute respiratory failure (*n* = 14, 53%) followed by shock (*n* = 5, 19%), perioperative management (*n* = 3, 12%), coma (*n* = 1, 4%) and acute renal failure (*n* = 1, 4%) (Table 1). Median SOFA score at admission was 7 [IQR, 5–8].

Only 3 patients (11%) had received prior antifungal prophylaxis effective against Mucorales. Mucormycosis was "proven" in 14 patients (54%), "probable" in 5 patients (19%) and “possible” in 7 patients (27%) in whom diagnosis was made by qPCR (Table 2). *Rhizopus* and *Mucor* were the most frequent documented species (*n* = 8, 31%), followed by *Lichteimia* (*n* = 3, 12%), *Rhizomucor* (*n* = 2, 8%) and *Lichteimia/Rhizomucor* (*n* = 1, 4%). Eight patients (31%) had concurrent fungal infection, related to *Aspergillus* spp (*n* = 7) and *Alternaria* spp (*n* = 1). Concurrent bacterial and viral infections were found in 8 and 4 patients, respectively. Serum galactomannan antigen was performed in 19 patients and was positive in 5 patients, including 4 with concurrent aspergillosis. Beta-D-glucane was positive in 1 (of 7 tested) patient, which experienced *Enterococcus faecium* bacteremia. Mucormycosis was diagnosed 1 day [−4 to + 6] after ICU admission. In 17 patients (65%), mucormycosis was diagnosed in ICU with a median time from admission of 4 days [IQR, 1–7]. In the remaining 9 patients, diagnosis was made before admission to ICU with a median time of 37 days [IQR, 4–60].

**Table 2:** Characteristics of mucormycosis and patients’ outcome

N (%) or Median (IQR)	All n = 26	Survivors in ICU n = 6	Non survivors in ICU n = 20	*P* value
***Diagnosis***^a^				0.12
Proven	14 (54)	2 (33)	12 (60)	
Probable	5 (19)	3 (50)	2 (10)	
Possible with positive qPCR	7 (27)	1 (17)	6 (30)	
***Involvement***				
Pulmonary	16 (62)	4 (67)	12 (60)	1.00
Rhino-cerebral	10 (38)	3 (50)	7 (35)	0.85
Digestive	5 (19)	0	5 (25)	0.44
Skin	2 (8)	0	2 (10)	1.00
Disseminated	8 (31)	1 (17)	7 (35)	0.73
***Species***				0.45
* Rhizopus/Mucor*	8 (31)	3 (50)	5 (25)	
* Lichteimia*	3 (12)	0	3 (15)	
* Rhizomucor*	2 (8)	0	2 (10)	
* Lichteimia/Rizomucor*	1 (4)	0	1 (5)	
* Rhizopus*	1 (4)	0	1 (5)	
* Mucor*	1 (4)	0	1 (5)	
Unknown	10 (38)	3 (50)	7 (35)	
***Concomitant infection***				
Fungal	8 (31)	3 (50)	5 (25)	1.00
Bacterial	8 (31)	2 (33)	6 (30)	0.51
Viral	4 (15)	0	4 (20)	0.59
***Specific treatments***				
Antifungal therapy				
- Curative L-AmB	22 (85)	6 (100)	16 (80)	0.59
- Initial dosage of curative L-AmB	5.0 [5.0–10.0]	10.0 [5.0–10.0]	5.0 [5.0–5.8]	0.86
- Maximal dosage of curative L-AmB	10.0 [5.5–10.0]	10.0 [8.1–10.0]	10.0 [5.0–10.0]	0.28
- Dual therapy	12 (46)	4 (67)	8 (40)	0.50
Curative surgery	8 (31)	2 (33)	6 (30)	1.00
***Outcomes***				
Length of ICU stay (days)	6.0 [2.3–10.8]	4.0 [1.8–5.5]	8.5 [2.8–17.5]	0.18
Length of hospital stay (days)	28.5 [21.0–41.3]	25.0 [15.0–37.0]	29.0 [22.0–44.5]	0.64
End-of-life decision	14 (54)	3 (50)	11 (90)	1.00

*ICU* intensive care unit, *L-AmB* liposomal amphotericin B

^a^According to EORTC/MSG criteria

Sixteen patients (62%) had pulmonary involvement whereas 10 (38%) had rhino-cerebral involvement (Table 2). Digestive tract and skin were involved in 5 (19%) and 2 (8%) patients respectively. Mucormycosis was disseminated in 8 patients (31%).

At CT scan, sinus involvement was always multiple, with a median of 3 sinuses affected [IQR, 3–3], associated with orbital involvement in 4 patients and cerebral involvement in 5 patients. Most patients with pulmonary invasion had multiple nodules (*n* = 8) or consolidations (*n* = 6), mainly associated with halo sign (*n* = 6) and sometimes with micronodules. Pleural effusion was associated in 63% of these cases.

Before diagnosis, 21 patients (81%) had received probabilistic antifungal therapy, including caspofungine (*n* = 9), liposomal amphotericin B (*n* = 5), voriconazole (*n* = 5) and fluconazole (*n* = 2). When mucormycosis diagnosis was established, 22 patients (85%) were treated with curative liposomal amphotericin B at a median dosage of 5 mg/kg [IQR, 5–10] (Table 2). Curative antifungal treatment was started with a median time of 1 day [− 4 to + 6] after ICU admission. Liposomal amphotericin B dosage was then increased in 8 patients (36%) at a maximal dosage of 10 mg/kg [IQR, 9–10]. Antifungal combination treatment was given to 12 patients (46%), as a salvage therapy in half of the cases, including posaconazole in 7, caspofungine in 2, voriconazole in 2 and isavuconazole in 1 patient. Eight patients (31%) underwent curative surgery. Among them, 4 patients had a disseminated mucormycosis, 2 had an isolated rhino-cerebral involvement, whereas the lung and digestive tract were solely involved in 1 patient each. Thirteen patients (50%) received granulocyte colony-stimulating factor.

Twenty-one patients (81%) required invasive mechanical ventilation (IMV). Median length of IMV was 5 days [IQR, 1–15]. Eighteen patients (69%) required vasopressors, mostly norepinephrine. Nine patients (35%) required renal replacement therapy.

In 14 patients (54%), end-of-life management was implemented in the ICU.

**Fig. 1:**
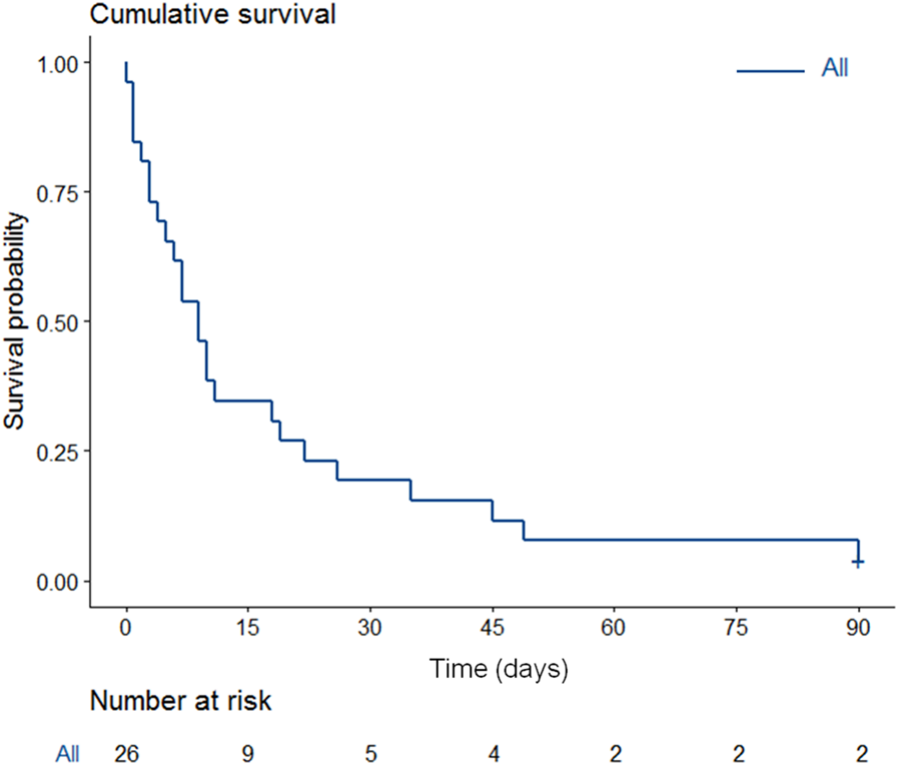
Kaplan–Meier curve estimates of overall survival in the whole cohort

ICU and hospital mortality rates were 77% and 88%, respectively. The median overall survival was 9 days [IQR, 3–22] (Fig. 1). Only two patients were alive at day 90.

IMV was strongly associated with ICU mortality (Table 1). After adjustment, three variables were associated with day 90 mortality namely allogeneic SCT (HR 4.84 [95% CI 1.64–14.32]), severity according to SOFA score (HR 1.19 per point [95% CI 1.02–1.39]) and use of dual therapy (HR 3.02 [95% CI 1.18–7.72]) (Table 3).

**Table 3:** Variables associated with day-90 mortality in multivariate (Cox model) analysis

Variables	Hazard ratio	95% confidence interval	*P* value
SOFA score	1.19	(1.02–1.39)	0.03
Dual therapy	3.02	(1.18–7.72)	0.02
Allogeneic SCT	4.84	(1.64–14.32)	0.004

*SOFA* sequential organ failure assessment, *SCT* stem cell transplantation

**Fig. 2:**
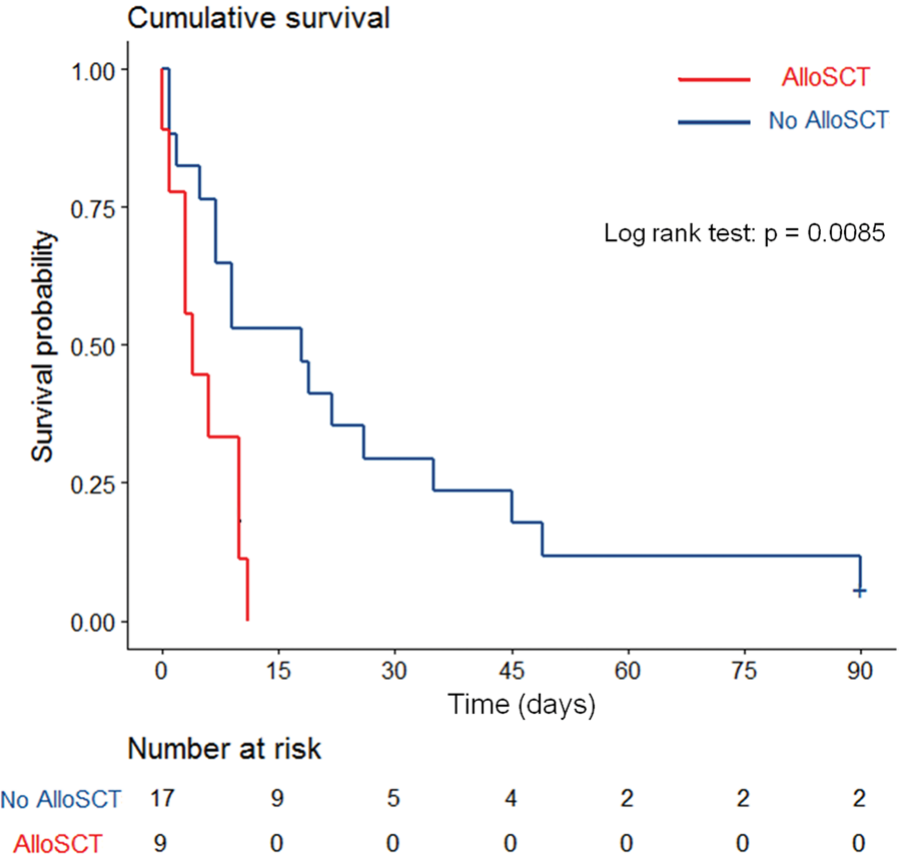
Kaplan–Meier curve reporting unadjusted influence of allogeneic stem cell transplantation (alloSCT). Kaplan–Meier curve estimates of overall survival in allogeneic stem cell transplant recipients (*n* = 9, red) and in other hematological patients (*n* = 17, blue). Survival between groups was compared using the Kaplan–Meier estimator. Univariate analysis was performed with the log rank test

Most of allogeneic SCT recipients (*n* = 9, 35%) were transplanted from matched unrelated donor and received peripheral blood stem cell after a myeloablative conditioning regimen. Acute leukemia was the most frequently indication of SCT. Median time between SCT and mucormycosis was 46 days [IQR, 14–158]. Four patients experienced GVHD at ICU admission, including two acute GVHD and two chronic GVHD requiring steroids. The median overall survival was 4 days [IQR, 3–10] (Fig. 2). All allogeneic SCT patients died in the ICU.

## Discussion

This multicenter study highlights that mucormycosis is associated with a high mortality rate in patients with HM admitted in the ICU, especially in allogeneic SCT recipients.

Mucormycosis is the third most common IFI in allogeneic SCT patients and is associated with an overall 1-year survival at 28% [4]. Moreover, allogeneic SCT patients admitted to the ICU with IFI (other than invasive aspergillosis) are at high risk of death [11]. In recent meta-analysis about allogeneic SCT patients admitted to the ICU, the number of organ failures, IMV and GVHD were associated with short-term mortality [11, 12] and can explain the high mortality rate in this subgroup in our cohort. However, organ replacement therapy rates in our study are higher than those previously reported [11] and probably reflect the severity of this IFI.

In a recent study of mucormycosis in critically ill patients, Claustre et al. showed that the presence of a hematological malignancy, in more than half of patients, was associated with higher mortality [13]. In this subgroup of patients, older age was associated with a poorer prognosis [13]. Although the median age was younger in our cohort, we reported here a higher ICU mortality rate and shorter overall survival. Those data highlight the dramatic prognosis of patients with HM and mucormycosis requiring ICU management.

The European Conference on Infections in Leukemia (ECIL) and the European Confederation of Medical Mycology (ECMM) [14] recently published guidelines for the management of mucormycosis: they both strongly support liposomal amphotericin B as a first-line treatment in adults. The initial recommended dose regimen is about 5 to 10 mg/kg. In the absence of CNS involvement, the use of 5 mg/kg was reported to be successfull. Patients who received the highest dose regimens tended to have a greatest rate of cure, despite more frequent renal adverse effects [15]. In our study, the patients received curative liposomal amphotericin B at a median dosage of 5 mg/kg [IQR, 5–10]. According to the latest guidelines, doses below 5 mg/kg are not recommended, especially in the most severe patients in the ICU. Data regarding the use of isavuconazole as a first-line therapy are scarce: in a case–control study, 21 patients with invasive mucormycosis received isavuconazole, and for 54% of them the disease was stable or in remission at day 42 [16]. Isavuconazole is not currently the gold standard in critically ill patients. It should be discussed in the presence of a contra-indication to liposomal amphotericin B or in case of salvage therapies.

Dual therapy was associated with a poorer outcome, probably in relation with a selection bias, being used in the most severe patients. Thus, half of them received antifungal combination treatment as a salvage therapy. Enhanced activity of liposomal amphotericin combined with micafungin or anidulafungin was demonstrated in mice with disseminated mucormycosis [17]. Nevertheless, available data failed to demonstrate a clear benefit in the treatment of mucormycosis [18]. To date, combination of antifungal treatements is not recommended as a first‐line therapy [14]. Further prospective studies are warranted to assess the value of combinations as a front-line therapy, as well as new antifungal therapies and dose adaptation according to species.

The latest ECIL guidelines recommend the use of antifungal prophylaxis, especially posaconazole, in patients with acute myeloid leukemia and allogeneic SCT recipients [19]. In our cohort, due to the large inclusion period (2002–2018), only 27% of patients received antifungal prophylaxis, which was effective against mucorales in 3. In the absence of a control population and available posaconazole dosages, our data do not allow us to conclude on potential efficacy of antifungal prophylaxis.

Although previous studies reported that surgical treatment decreased the risk of death [13, 20], this result was not confirmed in our cohort. Nevertheless, half of the patients who experienced surgical treatment in our cohort had a disseminated infection while it was established that surgery is mainly recommended for rhino-orbito-cerebral infection and soft tissue infection [21]. In this context, it seems difficult to conclude about the role of surgery in our cohort. In Claustre’s study, surgery was reported as a major prognostic factor in patients with HM [13]. However, this procedure was performed in only 29% of patients suggesting a strong selection of patients who could benefit from the intervention. Among them, 41% were alive at day 100. These late data prompt to temper the feasibility of surgery in this setting and its real impact.

An improvement of diagnostic methods and species identification is also needed: in our cohort, qPCR provided the diagnosis in nearly one third of the cases. Despite qPCR is not part of the EORTC criteria [7], those data may support its use to diagnose mucormycosis early and initiate specific treatment in a shorter time compared to histological or mycological evidence [22]. Moreover, qPCR seems also useful to monitor treatment and associated with a better survival in case of negativity after treatment initiation [22]. Further studies are warranted to assess the performances of qPCR and its impact in clinical practice.

## Conclusions

In conclusion, ICU management of invasive mucormycosis is associated with a high mortality in patients with HM. Benefit of ICU admission may deserve to be assessed individually and patients’ values taken into account. Improvement of diagnostic strategies, antifungal therapies and ICU management are urgently needed in this setting.

## Data Availability

The dataset supporting the conclusions of this article is included within the article (and its additional files).

## Abbreviations

ALL: Acute lymphoblastic leukemia
AML: Acute myeloid leukemia
CNS: Central nervous system
ECIL: European Conference on Infections in Leukemia
ECMM: European Confederation of Medical Mycology
EORTC/MSG: European Organization for Research and Treatment of Cancer/Mycoses Study Group
GVHD: Graft-versus-host disease
HM: Hematological malignancies
ICU: Intensive care unit
IFI: Invasive fungal infections
IMV: Invasive mechanical ventilation
L-AmB: Liposomal amphotericin B
qPCR: Quantitative polymerase chain reaction
SCT: Hematopoietic stem cell transplant
SOFA: Sepsis-related organ failure assessment
